# Exosome markers of LRRK2 kinase inhibition

**DOI:** 10.1038/s41531-020-00138-7

**Published:** 2020-11-13

**Authors:** Shijie Wang, Kaela Kelly, Jonathan M. Brotchie, James B. Koprich, Andrew B. West

**Affiliations:** 1 Department of Pharmacology and Cancer Biology, Duke Center for Neurodegeneration Research, Duke University, Durham, NC USA; 2 Atuka Inc., Toronto, ON Canada

**Keywords:** Parkinson's disease, Predictive markers

## Abstract

Hyper-activated LRRK2 is linked to Parkinson’s disease susceptibility and progression. Quantitative measures of LRRK2 inhibition, especially in the brain, maybe critical in the development of successful LRRK2-targeting therapeutics. In this study, two different brain-penetrant and selective LRRK2 small-molecule kinase inhibitors (PFE-360 and MLi2) were orally administered to groups of cynomolgus macaques. Proposed pharmacodynamic markers in exosomes from urine and cerebrospinal fluid (CSF) were compared to established markers in peripheral blood mononuclear cells (PBMCs). LRRK2 kinase inhibition led to reductions in exosome-LRRK2 protein and the LRRK2-substrate pT73-Rab10 in urine, as well as reduced exosome-LRRK2 and autophosphorylated pS1292-LRRK2 protein in CSF. We propose orthogonal markers for LRRK2 inhibition in urine and CSF can be used in combination with blood markers to non-invasively monitor the potency of LRRK2-targeting therapeutics.

## Introduction

Leucine-rich repeat kinase 2 (LRRK2) small-molecule kinase inhibitors and anti-sense oligonucleotides have demonstrated neuroprotective efficacy in some models of Parkinson’s disease (PD) and represent a promising novel class of disease-modifying therapeutics for neurodegeneration^1–3^. Efforts to develop potent and selective therapies that mitigate LRRK2-hyperactivation associated with pathogenic *LRRK2* mutations have recently intensified in industry^4,5^. Reliable measures of LRRK2 expression and enzymatic inhibition may assist in the identification of the most efficacious LRRK2-targeting therapies, particularly in early phase clinical trials^6,7^.

LRRK2 exists as a phospho-protein in all cells and tissues so-far evaluated^7,8^, with an N-terminal cluster of phosphorylated residues sensitive to LRRK2 kinase inhibition^9–11^. The more recently discovered LRRK2-autophosphorylation residue pS1292-LRRK2 localizes to the C-terminal region of the LRRK2 leucine-rich repeat (LRR) domain, nearby the Rab-like ROC domain^12–14^. LRRK2 kinase activity also regulates levels of pT73-Rab10, as observed in inhibitor treatments of immune cells that express high levels of LRRK2 protein (e.g., monocytes, neutrophils)^15^. Inhibition of pT73-LRRK2 has also been observed in tissues (e.g., lung and kidney) procured from mice and rats treated with LRRK2 kinase inhibitors^6,16,17^.

As some LRRK2-targeting molecules have progressed through initial safety trials, non-invasive pharmacodynamic LRRK2-inhibition measures in biofluids maybe valuable to establish on-target drug effects across the body, particularly in the brain. Recently, a preclinical study in macaques recorded reductions of pS935-LRRK2 in brain tissue and peripheral blood mononuclear cells (PBMCs) in response to different doses of efficacious small-molecule LRRK2 kinase inhibitors including PFE-360 and MLi2^18^. Reductions in the LRRK2 kinase substrate pT73-Rab10 were also observed in lung tissue. A study in mice measuring pS1292-LRRK2 (autophosphorylated) levels in response to MLi2 treatment found reductions in both brain and lung tissues^19^. While it would not be practical to directly obtain brain or lung tissue in the course of a clinical trial to establish on-target drug effects of inhibitors, previously, we identified LRRK2 protein in different biofluids localizing to exosome microvesicles^20^. Through the procurement of biofluids, exosomes could be potentially utilized for monitoring LRRK2-target engagement. We have recently postulated that exosome-LRRK2 and exosome-Rab proteins may represent a valuable source for pharmacodynamic biomarkers to better understand LRRK2-target engagement profiles among different therapeutics^7^.

Herein, we measured exosome-harbored LRRK2 and Rab10 proteins for pharmacodynamic responses to LRRK2 kinase inhibition in macaques treated with the small-molecule inhibitors PFE-360 and MLi2. Our data provide suggest that exosome markers in urine and cerebrospinal fluid (CSF) may complement other markers in blood cells in a highly informative panel to establish on-target LRRK2 inhibition and efficacious doses of drugs in the clinic.

## Results

### PBMC markers of LRRK2 kinase inhibition

To measure pharmacodynamic responses of LRRK2 kinase inhibition in macaques, PBMCs, plasma, urine, and CSF were procured from ten animals prior to drug administration. Two groups of five macaques were then treated daily with either 5 mg kg^−1^ of PFE-360 or MLi2. On the fifth day of treatment, biofluids were collected two hours after the final drug dose to achieve unbound drug plasma profiles within the range of a previous report where brain tissue was analyzed for LRRK2 inhibition (i.e., pS935-LRRK2)^18^. At the time of biofluid collection, drug plasma concentrations of PFE-360 varied as expected in the five macaques from ~40 to ~145 nM, whereas MLi2 produced drug plasma concentrations that varied between ~12 and ~70 nM. No adverse drug reactions were noted in any animal during the treatment period.

**Fig. 1:**
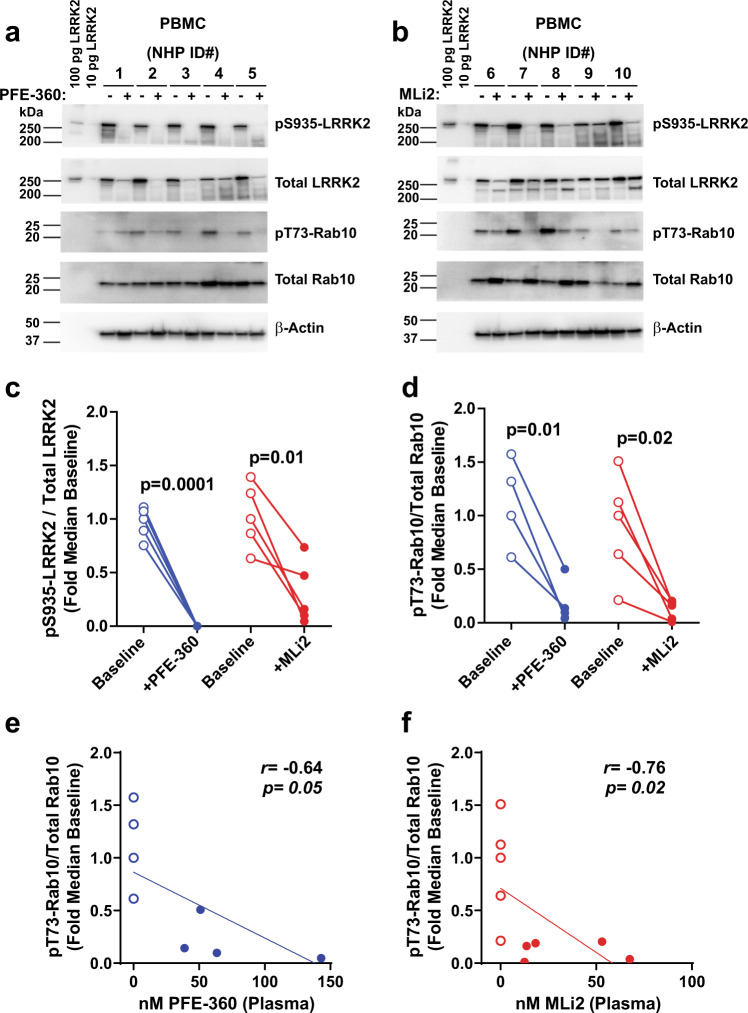
LRRK2 kinase inhibition in PBMCs. Representative western blots for the indicated proteins in PBMCs isolated from NHPs at baseline and after 5 days of **a** PFE-360 and **b** MLi2 treatment (each 5 mg kg^−1^ QD, whole blood collected 2-h post-dose). Recombinant LRRK2 protein standards (100 and 10 pg) were utilized as positive controls for LRRK2 antibody detection. Fold median baseline values before and after PFE-360 (blue) or MLi2 (red) treatment for **c** pSer935-LRRK2 levels normalized to total LRRK2 protein and **d** pT73-Rab10 levels normalized total Rab10 protein. Open circles are baseline measurements and closed circles are plasma drug exposures 2 h post-final dose. Paired *t*-tests were performed to determine the significance between baseline and post-drug exposures. Scatterplots of pT73-Rab10/Total Rab10 protein for **e** PFE-360 (nM) and **f** MLi2 (nM). Spearman’s rank correlation (*r*_s_) was used to evaluate associations between plasma drug levels and reductions in pT73-Rab10/Total Rab10 protein levels. For Rab10 inhibition measures (**d**–**f**), one outlier sample in the PFE-360 treatment group was not included because baseline pT73-Rab10 levels fell below the limit of detection (**a** NHP ID# 1).

LRRK2 is differentially expressed in immune cells in PBMCs, with some of the highest LRRK2 expression in monocytes^21–24^. Similar to Baptista et al.^18^, pS935-LRRK2 to total LRRK2 ratios were dramatically reduced with both PFE-360 and MLi2 treatment compared to baseline levels (Fig. 1A–C). PFE-360 treated macaques had no measurable pS935-LRRK2 levels compared to baseline collections from the same animals (Fig. 1C). However, MLi2 treatment did not result in a complete loss of pS935-LRRK2 protein, with the signal detected in all MLi2-treated macaques (albeit at low levels). One macaque showed only a 37% reduction in pS935-LRRK2 that also had the lowest concentration of unbound plasma MLi2 (12.5 nM, Fig. 1C). Although the LRRK2-autophosphorylation site pS1292 has been detected in human urinary and CSF exosomes in past studies, pS1292-LRRK2 was not reliably detected in the PBMC lysates we procured from the macaques.

Measures of the ratio of pT73-Rab10 to total Rab10 revealed nearly comparable reductions as that observed for pS935-LRRK2 levels with drug treatment. Four of five macaques treated with PFE-360 demonstrated a robust reduction in pT73-Rab10 levels, where one outlying macaque had baseline levels of pT73-Rab10 that fell below our limit of detection and was excluded (Fig. 1A, NHP ID# 1). Otherwise, LRRK2 *trans-*phosphorylation of the pT73-Rab10 substrate was successfully detected at low levels in PBMCs, where on average, pT73-Rab10 levels were reduced by 77 and 87% post-PFE-360 and MLi2 exposure, respectively (Fig. 1D). Furthermore, significant correlations between plasma drug levels and pT73-Rab10 levels in PBMCs were identified for both compounds (PFE-360 *r*_s_(7) = −0.64, *p* = 0.05; MLi2 *r*_s_(9) = −0.76, *p* = 0.02). Overall, higher plasma drug concentrations correlated with greater reductions in pT73-Rab10 levels (Fig. 1E, F). These results demonstrate that pT73-Rab10 provides a second pharmacodynamic biomarker in PBMCs, supporting past results that demonstrate ex vivo treatment of human PBMCs with MLi2 ablate pT73-Rab10 levels and pS935-LRRK2 levels^25^.

### Urine markers of LRRK2 kinase inhibition

**Fig. 2:**
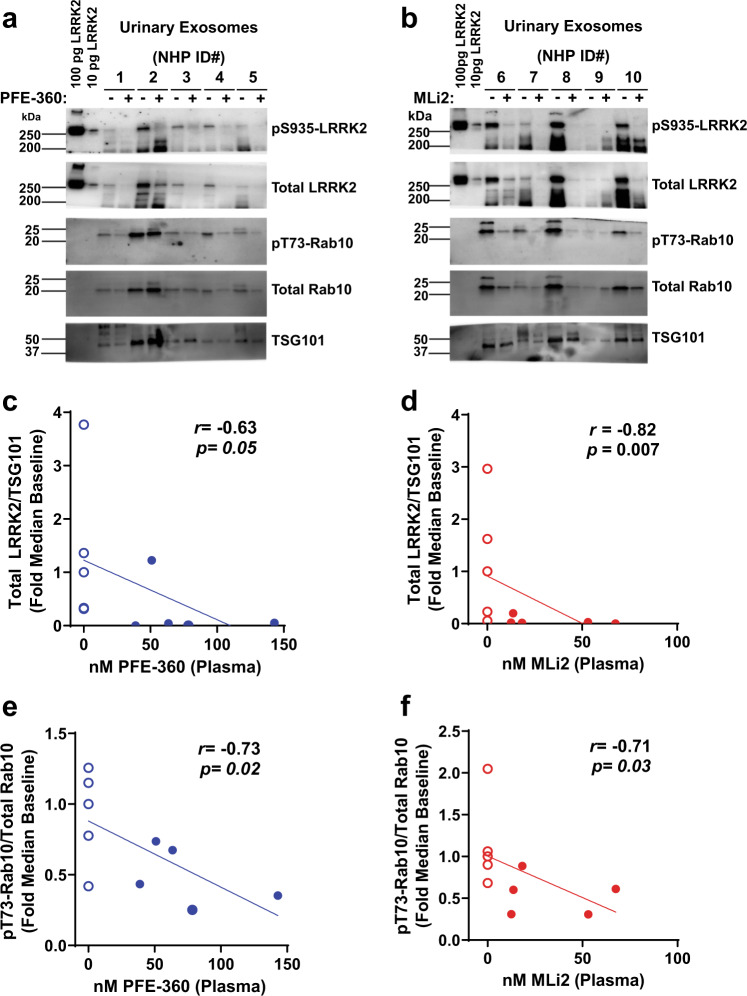
LRRK2 inhibition in urinary exosomes. Representative western blots for LRRK2 and Rab10 proteins in urinary exosomes procured from NHPs at baseline and after 5 days of **a** PFE-360 and **b** MLi2 treatment (5 mg kg^−1^ QD). Recombinant LRRK2 protein standards (100 and 10 pg) were utilized as positive controls for LRRK2 antibody detection. Scatterplots of plasma drug levels for **c** PFE-360 (nM) or **d** MLi2 (nM) and total LRRK2 protein levels. Total LRRK2 protein isolated from urinary exosomes were normalized to the exosome marker TSG101. Scatterplots of plasma drug levels for **e** PFE-360 (nM) or **f** MLi2 (nM) and pT73-Rab10/total Rab10 levels. Open circles are baseline measurements and closed circles are plasma drug exposures 2 h post-final dose. Spearman’s rank correlation (*r*_s_) was used to evaluate associations between plasma drug levels and reductions in exosomal total LRRK2 protein or pT73-Rab10/total Rab10 levels.

Our previous work localized LRRK2 protein expression in urine to a type of microvesicle called exosomes^26,27^. Urinary exosomes contain protein from tissues throughout the body without apparent enrichment of kidney or urine proteins^27^. Some evidence in cell lines suggests that LRRK2 kinase inhibitor treatment may block the release of LRRK2 into exosomes^20^. Similar to PBMCs, the LRRK2-autophosphorylation site pS1292-LRRK2 was not reliably detected in baseline macaque urine in our assay. PFE-360 or MLi2 treatment caused a profound decrease in total LRRK2 protein normalized to the exosome housekeeping protein TSG101 in all animals (PFE-360 *r*_s_(9) = −0.63, *p* = 0.05; MLi2 *r*_s_(9) = −0.82, *p* = 0.007, Fig. 2A–D). Although clear reductions were also noted with pS935-LRRK2 levels (Fig. 2A, B), owing to the stark reductions in total LRRK2 protein caused by kinase inhibition, the ratio of pS935-LRRK2 to total LRRK2 protein was uninformative. These results support past observations made in cell lines whereby LRRK2 kinase inhibitors reduce exosome-LRRK2 release^20^, although we cannot exclude cellular LRRK2 reductions of LRRK2 in different tissues throughout the body that contribute to urinary exosome pools of LRRK2 protein. Ratios of phospho-LRRK2 to total LRRK2 as a pharmacodynamic measure in urine could be considered confounded due to variable reductions in total LRRK2 protein levels.

Unlike LRRK2, total Rab10 levels did not appear to vary with drug treatment (Fig. 2E, F). Dose-dependent decreases in the ratio of pT73-Rab10 to total Rab10 protein were observed (PFE-360 *r*_s_(9) = −0.73, *p* = 0.02; MLi2 *r*_s_(9) = −0.71, *p* = 0.03), where the greatest reductions occurred in the macaques with the highest plasma concentrations of the drug. Notably, the reduction of pT73-Rab10 was incomplete with the detectable signal in all samples found, even with higher plasma concentrations of drug and a near-complete loss of signal in PBMCs for the same markers (Fig. 1). These results support measures of pT73-Rab10 in urine as a novel pharmacodynamic marker for LRRK2 kinase inhibition without an apparent floor-effect of the marker associated with higher levels of drug.

### Cerebrospinal fluid markers of LRRK2 kinase inhibition

**Fig. 3:**
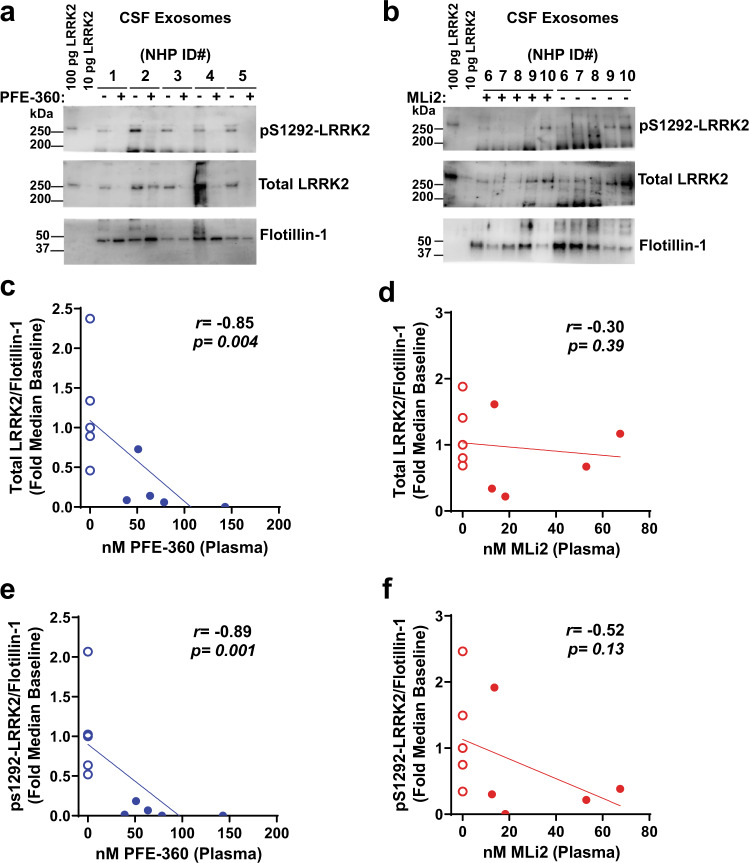
LRRK2 inhibition in exosomes isolated from CSF. Representative western blots for LRRK2 proteins in CSF exosomes procured from NHPs at baseline and after 5 days of **a** PFE-360 and **b** MLi2 treatment (5 mg kg^−1^ QD). Recombinant LRRK2 protein standards (100 and 10 pg) were utilized as positive controls for LRRK2 antibody detection. Scatterplots of plasma drug levels for **c** PFE-360 (nM) or **d** MLi2 (nM) and total LRRK2 protein levels. Total LRRK2 protein isolated from CSF exosomes were normalized to the exosome marker Flotillin-1. Scatterplots of plasma drug levels for **e** PFE-360 (nM) or **f** MLi2 (nM) and pS1292-LRRK2 protein normalized to Flotillin-1. Open circles are baseline measurements and closed circles are plasma drug exposures 2 h post-final dose. Spearman’s rank correlation (*r*_s_) was used to evaluate associations between plasma drug levels and reductions in exosomal total LRRK2 protein or pS1292-LRRK2 levels.

Previously we demonstrated that LRRK2 protein and pS1292-LRRK2 protein, but not Rab10 protein, can be readily detected and measured in exosome fractions from biobanked human CSF using our assays^12^. In CSF exosomes, LRRK2-autophosphorylation at the serine 1292 residue is much higher (~5-fold increased on average) as compared to urinary exosomes collected from the same human subjects^12^. In macaque CSF exosomes (Fig. 3A, B), total LRRK2 levels diminished with PFE-360 treatment (*r*_s_(9) = −0.85, *p* = 0.004; Fig. 3C), but not with MLi2 treatment (*r*_s_(9) = −0.30, *p* = 0.39; Fig. 3D). These results are consistent with past observations in brain tissue made with rats and mice treated PFE-360 and MLi2, where PFE-360 had a larger effect on destabilizing LRRK2 protein at the same drug concentrations as MLi2^6^.

Similar reductions were also observed for exosomal pS1292-LRRK2 levels with PFE-360 treatment (*r*_s_(9) = −0.89, *p* = 0.01; Fig. 3E), while MLi2 treatment reduced pS1292-LRRK2 levels in four of the macaques with one macaque not responding (*r*_s_(9) = −0.52, *p* = 0.13; Fig. 3F). Both total LRRK2 and pS1292-LRRK2 levels were normalized to the exosome housekeeping protein flotillin-1 to account for any possible differences in exosome isolation efficiency and lysis efficiency. TSG101, used in urine, appears much less abundant in CSF compared to flotillin. Because of the possible decrease observed with both inhibitors in total LRRK2 protein, ratios of pS1292-LRRK2 to total LRRK2 protein were not calculated (as total LRRK2 levels approached zero in several samples). In CSF exosome lysates from all samples, pS935-LRRK2, pT73-Rab10, and total Rab10 could not reliably be detected or measured, presumably due to lower abundance of these proteins compared to urine exosome and PBMC samples. Overall, these results support the feasibility of measures of total LRRK2 and pS1292-LRRK2 in CSF and support their possible utility as pharmacodynamic biomarkers for LRRK2-targeting therapeutics (i.e., small-molecule inhibitors and anti-sense oligonucleotides).

## Discussion

**Fig. 4:**
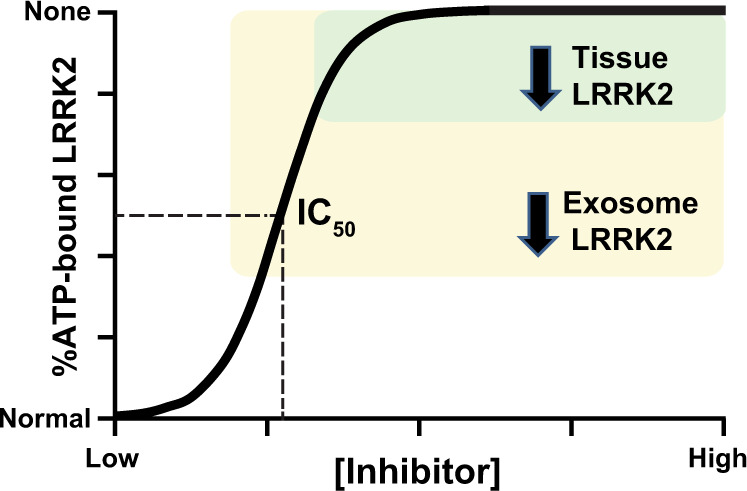
Hypothesis for extracellular and cellular LRRK2 inhibition. Diagram illustrating a hypothetical relationship of LRRK2 protein in tissue or biofluids (i.e., in exosomes), with %ATP-bound LRRK2 with increasing concentrations of ATP-competitive small-molecule LRRK2 kinase inhibitors. In the model, exosome depletion of LRRK2 occurs initially without lowering tissue levels of total LRRK2 is shown in yellow. The green area highlights higher concentrations of drug and the corresponding loss of any ATP-bound LRRK2, resulting in the depletion of LRRK2 protein in both exosomes and tissues.

This study identifies efficacious target engagement measures for LRRK2 kinase inhibition in exosomes isolated from urine and CSF of non-human primates using two distinct small-molecules (PFE-360 and MLi2). Our results build on observations comparing pS935-LRRK2 PBMC and brain tissue lysate measurements in treated macaques^18^, as well as pS1292-LRRK2 levels measured in treated mice^19^. Consistent with our past studies in transfected cell lines^20^, exosome levels of total LRRK2 protein are diminished with acute small-molecule LRRK2 inhibitor exposures. Reductions of pT73-Rab10 measured in urinary exosomes provide an additional informative measure. We provide evidence that measures of LRRK2 and pS1292-LRRK2 in CSF may provide a window into LRRK2 kinase inhibition in the brain. As both urine and CSF are readily procurable in the clinic, our data overall support the feasibility of panel-based integration of several pharmacodynamic biomarkers that might provide a better overall ascertainment of LRRK2 inhibition across the body. In consideration of this study and past studies in other model systems, we propose a relationship whereby reductions in exosomal LRRK2 protein occurs with LRRK2 kinase inhibition, sensitive to both inhibition-induced reduction of secretion as well as reductions of cellular LRRK2 caused by some kinase inhibitors (Fig. 4). Even in the context of reduced cellular levels of LRRK2 protein caused by inhibitor-induced destabilization, reductions in total LRRK2 can be measured in exosome pools when normalized against other housekeeping proteins like TSG101 and flotillin-1 to develop high-confidence assays and interpretations.

Previously, we and others demonstrated elevated LRRK2-autophosphorylation and LRRK2 protein levels associated with PD susceptibility and LRRK2 pathogenic mutations^12,26^. With high endogenous LRRK2 expression in some types of innate immune cells, PBMCs have been championed as a source for pharmacodynamic biomarkers related to LRRK2 inhibition^15^, and are currently utilized in clinical trials. However, measures in PBMCs alone may poorly predict LRRK2 inhibition in the brain and other tissues, for example with drugs that are poorly brain-penetrant or are rapidly eliminated. Drug concentration measurements in CSF are often erroneously conflated with brain tissue exposure and target engagement. PBMCs can also be difficult to interpret, as they rapidly turn over and transiently respond to diverse stimuli unrelated to the inhibitor treatment, affecting LRRK2 expression and phosphorylation. Further, our previous study suggests that the efficiency of LRRK2 inhibition can be tissue-specific, and brain LRRK2 protein maybe more resilient to inhibition compared to other tissues^6^. Ideally, we suggest that a panel of predictive markers that assess LRRK2 inhibition and pharmacodynamic responses across the body will better highlight the most efficacious drugs and dosage regimens. We suggest that measuring CSF levels of LRRK2 protein in exosomes could contribute to establishing brain penetration in early phase clinical trials (e.g., acute safety studies). Alternatively, even if target engagement assays are not woven into clinical trial design, as exosomes are exceedingly stable sources of proteins in biobanked biofluids, post-hoc measurements can be conducted to help interpret clinical trial results.

Although proteomic studies using mass spectrometry have suggested the presence of Rab proteins in the CSF, we have not yet detected pT73-Rab10 in the exosome fraction using our assay^28^. It is unclear whether this challenge is due to the low assay sensitivity or limited sample volumes. It is plausible that the inhibition profile of each drug would further change under different dosages or time intervals post-oral gavage, or in a chronic inhibition study such as an efficacy trial in the clinic. Further, this study did not evaluate the possible effects of age or sex on these markers, nor explore the normal biology of the markers in the biofluids. In comparing observations in PMBCs to CSF exosomes, our results here support our past results in mice and rats demonstrating the superiority of PFE-360 compared to MLi2 in inhibiting LRRK2 in brain tissue, even though peripheral LRRK2-inhibition potency is comparable between the two molecules^6^. Likewise, we would anticipate these assays maybe able to identify more potent drugs and concentrations in clinical trials.

Many new compounds have high attrition rates due to a presumed lack of target engagement at doses resolved in safety studies^29^. Implementation of informative pharmacodynamic panels of markers may help improve drug development outcomes. As potential LRRK2-targeting therapeutics like small-molecule LRRK2 kinase inhibitors and LRRK2-targeting anti-sense oligonucleotides move forward, there is an acute need for pharmacodynamic and theragnostic markers to monitor the success of different classes of LRRK2 kinase inhibitors in blocking LRRK2 activity associated with disease^7^. Notably, the protein measurement assays presented here are based on immunoblotting procedures that, while linear and quantitative in our protocols^12^, do not scale well into clinically available tests. Further assay development will be needed for broad clinical implementation and validation^7^.

Pharmacodynamic markers for LRRK2 inhibition have largely focused on measures of the ratio of pS935-LRRK2 to total LRRK2 protein and the ratio of pT73-Rab10 to total Rab10 protein in protein lysates from different tissues. Focusing on clinically available biofluids in non-human primates (macaques), we evaluate different combinations of pharmacodynamic markers for LRRK2-inhibition signatures in exosomes isolated from urine and CSF and how these different markers compare to LRRK2 inhibition measured in blood at the same time. Quantitative assessments for LRRK2 inhibition in urine and CSF, including the inhibition of LRRK2 secretion in exosomes, provide an inclusive evaluation of LRRK2-targeting therapeutics across the body. These results demonstrate the utility of readily procured biofluids (i.e., blood, urine, and CSF) in measuring LRRK2 inhibition in both the brain and periphery.

## Methods

### Compounds

Small-molecule LRRK2 kinase inhibitors PFE-360 and MLi2 were synthesized in-house as previously described^6^. Identity and high purity (i.e., >97.4% for each compound) were confirmed by nuclear-magnetic resonance and mass spectrometry. PFE-360 was originally discovered as part of a Pfizer chemistry program and MLi2 was originally discovered as part of a Merck chemistry program^30–32^. Previously assessed in vitro LRRK2 kinase assays show similar IC_50_ potencies with the two (structurally distinct) inhibitors, with PFE-360 demonstrating 6 nM IC_50_ inhibition and MLi2 demonstrating 4 nM inhibition for WT-LRRK2-autophosphorylation^6^. Both compounds show excellent selectivity, with off-target inhibition (kinases other than LRRK2) of IC_50_ < 500 nM for only two other known kinases (MST-2, MAP3K5) for PFE-360 and three other kinases for MLi2 (MAP3K14, CLK4, CLK2)^15,30,31^. No off-target interactions are known for either compound below 100 nM.

### Animals and drug doses

All husbandry, housing, and experimental procedures were conducted at Suzhou Xishan Zhongke Drug R&D Co., Ltd. (Suzhou, PRC) and under an IACUC animal use protocol specific for this study and approved by Suzhou Xishan Zhongke Drug R&D Co., Ltd. All methods were performed in accordance with the relevant guidelines and regulations. Female cynomolgus macaques averaged 10 years of age (Xishan, PRC), were orally administered 5 mg kg^−1^ PFE-360 or MLi2 daily for 5 days (*N* = 5 per group, 10 total). Drug doses were selected based on pharmacokinetic profiles and observations in a recent report, where 5 mg kg^−1^ PFE-360 or MLi2 produce unbound plasma exposures in macaques that extrapolate to 4.7-fold exposure (PFE-360) and 6.3-fold exposure (MLi2)-IC_50_ concentrations at C_max_ for LRRK2 inhibition in the mouse brain^18^. Under anesthesia (Zoletil/atropine (6/0.04 mg kg^−1^, IM)), blood, urine (via acute catheterization), and CSF (from cisterna magna), were collected before treatment (baseline), and again two hours post-final dose as described^18^. Proposed pharmacodynamic biomarkers for LRRK2 inhibition were comparisons of baseline levels to those levels after drug treatment.

### Blood measures

Macaque blood samples were collected into K2-EDTA vacutainer tubes and PBMCs isolated as described^18^. For determination of unbound compound concentrations, plasma was diluted 1:4 in acetonitrile, vortexed, and centrifuged at 4700×*g* for 15 min to precipitate protein. Drug plasma concentrations were measured from the resulting supernatant. Compound standards included 50% acetonitrile in water with known concentrations of compound (1–10,000 ng mL^−1^) spiked into macaque plasma. Standards and experimental solutions were analyzed on an Applied Biosystems Sciex Triple Quad 5500, with online LC-30 CE and Phenomenex Synergi 2.5 μm Polar-RP 3 × 50 mm columns. The mobile phase included 5% acetonitrile in water with 0.1% Formic acid and a solution of 95% acetonitrile in water with 0.1% formic acid.

### Protein measurements

PBMCs were lysed in Laemmli buffer (4% SDS, 10% glycerol, 120 mM Tris pH 6.8, 40 mM NaF) freshly supplemented with 50 mg mL^−1^ DTT (dithiothreitol). After 1-h incubation at 4 °C, lysates were centrifuged for 10 min at 4 °C at 15,000×*g*. Primary antibodies (all used at 1:1000, or ~0.5 to 1.0 μg mL^−1^) used include anti-LRRK2 (N241A/34, Antibodies Inc), anti-LRRK2 (clone MJFF2 c41-2, Abcam), anti-pS935-LRRK2 (clone UDD2-10, Abcam), anti-pS1292-LRRK2 (clone MJFR-19-7-8, Abcam), anti-pT73 Rab10 antibody (clone MJF-R21, Abcam), anti-Rab10 (clone D36C4, Cell Signaling), anti-flotillin-1 (clone D2V7J, Cell Signaling), and anti-TSG101 (ab30871, Abcam). The following secondary antibodies (1:10,000) were used: HRP conjugated donkey anti-rabbit secondary antibody (Jackson ImmunoResearch, # 711-035-152) and HRP conjugated donkey anti-mouse secondary antibody (Jackson ImmunoResearch, # 715-035-151). Immunoblots were developed using Luminata Crescendo Western HRP Substrate and digital signals were imaged and analyzed using ChemiDoc Imaging system (BioRad) and ImageLab software (BioRad). Assay linearity and variably associated with each measure in the different biofluids were previously reported^6,12,26^. Standard curves with recombinant protein demonstrated linearity between 10 pg to 500 pg for total LRRK2 and 2.8–140 pg for pS1292-LRRK2 to (*r* > 0.9) as shown previously^12^. Recombinant phospho-Rab protein standards were not available for this study. Repeated measure analysis of four biofluid samples from macaques from the starting biofluid volumes showed a coefficient of variation (CV) better than 20% for each assay. All immunoblots are derived from the same experiment and were processed in parallel.

### Exosome isolation

Urine (1 mL) and CSF samples (200 µL) were stored at −80 °C in cryo-vials (Corning) and quickly thawed in a water bath at 42 °C before processing. Fluids were centrifuged at 10,000×*g* for 30 min and the supernatants were transferred to polycarbonate centrifuge tubes. Samples were then centrifuged at 150,000×*g* for 2 h at 4 °C. Pellets (enriched in exosomes) were lysed in Laemmli buffer (as described above), and stored at −80 °C. Consistent with past approaches, TSG101 was selected as an exosome housekeeping protein and loading control in urinary exosomes, whereas flotillin-1 was selected as an exosome housekeeping protein and loading control in CSF-derived exosomes, owing to the relative differential abundance of each protein in the respective biofluid^12^.

### Statistical analysis

All described statistical analyses were performed using GraphPad Prism 8.0.

### Reporting summary

Further information on research design is available in the Nature Research Reporting Summary linked to this article.

## Supplementary information

Supplemental Figure

Reporting Summary

## Data Availability

The data sets generated and/or analyzed during the current study are available from the corresponding author on reasonable request.

## References

[1] Daher JP (2015). Leucine-rich repeat kinase 2 (LRRK2) pharmacological inhibition abates α-synuclein gene-induced neurodegeneration. J. Biol. Chem..

[2] Volpicelli-Daley LA (2016). G2019S-LRRK2 expression augments alpha-synuclein sequestration into inclusions in neurons. J. Neurosci..

[3] Zhao HT (2017). LRRK2 antisense oligonucleotides ameliorate α-synuclein inclusion formation in a Parkinson’s disease mouse model. Mol. Ther. Nucleic Acids.

[4] Alessi DR, Sammler E (2018). LRRK2 kinase in Parkinson’s disease. Science.

[5] West AB (2017). Achieving neuroprotection with LRRK2 kinase inhibitors in Parkinson disease. Exp. Neurol..

[6] Kelly K (2018). The G2019S mutation in LRRK2 imparts resiliency to kinase inhibition. Exp. Neurol..

[7] Kelly, K. & West, A. B. Pharmacodynamic biomarkers for emerging LRRK2 therapeutics. *Front. Neurosci.* **14**, 10.3389/fnins.2020.00807 (2020). 10.3389/fnins.2020.00807 PMC7438883 32903744

[8] West AB (2007). Parkinson’s disease-associated mutations in LRRK2 link enhanced GTP-binding and kinase activities to neuronal toxicity. Hum. Mol. Genet.

[9] Dzamko N (2010). Inhibition of LRRK2 kinase activity leads to dephosphorylation of Ser(910)/Ser(935), disruption of 14-3-3 binding and altered cytoplasmic localization. Biochem. J..

[10] Li X (2011). Phosphorylation-dependent 14-3-3 binding to LRRK2 is impaired by common mutations of familial Parkinson’s disease. PLoS ONE.

[11] Nichols RJ (2010). 14-3-3 binding to LRRK2 is disrupted by multiple Parkinson’s disease-associated mutations and regulates cytoplasmic localization. Biochem. J..

[12] Wang S (2017). Elevated LRRK2 autophosphorylation in brain-derived and peripheral exosomes in LRRK2 mutation carriers. Acta Neuropathol. Commun..

[13] Liu Z (2018). LRRK2 phosphorylates membrane-bound Rabs and is activated by GTP-bound Rab7L1 to promote recruitment to the trans-Golgi network. Hum. Mol. Genet..

[14] Henry AG (2015). Pathogenic LRRK2 mutations, through increased kinase activity, produce enlarged lysosomes with reduced degradative capacity and increase ATP13A2 expression. Hum. Mol. Genet..

[15] Thirstrup K (2017). Selective LRRK2 kinase inhibition reduces phosphorylation of endogenous Rab10 and Rab12 in human peripheral mononuclear blood cells. Sci. Rep..

[16] Lis P (2018). Development of phospho-specific Rab protein antibodies to monitor in vivo activity of the LRRK2 Parkinson’s disease kinase. Biochem. J..

[17] Fan Y (2018). Interrogating Parkinson’s disease LRRK2 kinase pathway activity by assessing Rab10 phosphorylation in human neutrophils. Biochem. J..

[18] Baptista MAS (2020). LRRK2 inhibitors induce reversible changes in nonhuman primate lungs without measurable pulmonary deficits. Sci. Transl. Med..

[19] Kluss JH (2018). Detection of endogenous S1292 LRRK2 autophosphorylation in mouse tissue as a readout for kinase activity. NPJ Parkinsons Dis..

[20] Fraser KB (2013). LRRK2 secretion in exosomes is regulated by 14-3-3. Hum. Mol. Genet..

[21] Hakimi M (2011). Parkinson’s disease-linked LRRK2 is expressed in circulating and tissue immune cells and upregulated following recognition of microbial structures. J. Neural Transm..

[22] Thévenet J, Pescini Gobert R, Hooft van Huijsduijnen R, Wiessner C, Sagot YJ (2011). Regulation of LRRK2 expression points to a functional role in human monocyte maturation. PLoS ONE.

[23] Cook DA (2017). LRRK2 levels in immune cells are increased in Parkinson’s disease. npj Parkinson’s Dis..

[24] Kubo M (2010). LRRK2 is expressed in B-2 but not in B-1 B cells, and downregulated by cellular activation. J. Neuroimmunol..

[25] Ito G (2016). Phos-tag analysis of Rab10 phosphorylation by LRRK2: a powerful assay for assessing kinase function and inhibitors. Biochem. J..

[26] Fraser KB, Moehle MS, Alcalay RN, West AB (2016). Urinary LRRK2 phosphorylation predicts parkinsonian phenotypes in G2019S LRRK2 carriers. Neurology.

[27] Wang S, Kojima K, Mobley JA, West AB (2019). Proteomic analysis of urinary extracellular vesicles reveal biomarkers for neurologic disease. EBioMedicine.

[28] Chiasserini D (2014). Proteomic analysis of cerebrospinal fluid extracellular vesicles: a comprehensive dataset. J. Proteom..

[29] Morgan P (2012). Can the flow of medicines be improved? Fundamental pharmacokinetic and pharmacological principles toward improving phase II survival. Drug Discov. Today.

[30] Scott JD (2017). Discovery of a 3-(4-pyrimidinyl) indazole (MLi-2), an orally available and selective leucine-rich repeat kinase 2 (LRRK2) inhibitor that reduces brain kinase activity. J. Med. Chem..

[31] Fell MJ (2015). MLi-2, a potent, selective, and centrally active compound for exploring the therapeutic potential and safety of LRRK2 kinase inhibition. J. Pharm. Exp. Ther..

[32] Henderson, J. L. et al. Discovery and preclinical profiling of 3-[4-(morpholin-4-yl)-7H-pyrrolo[2,3-d]pyrimidin-5-yl]benzonitrile (PF-06447475), a highly potent, selective, brain penetrant, and in vivo active LRRK2 kinase inhibitor. *J. Med. Chem.* **58**, 419–432 (2015). 10.1021/jm5014055 25353650

