# Supplementary material for: Exosome markers of LRRK2 kinase inhibition
Source: NPJ Parkinsons Dis. 2020 Nov 13;6:32. doi: 10.1038/s41531-020-00138-7 (PMC7666125; doi:10.1038/s41531-020-00138-7)
Supplement: Supplementary file 1 — Supplemental Figure [file 41531_2020_138_MOESM1_ESM.pdf]

Exosome Markers of LRRK2 Kinase Inhibition

Shijie Wang[1++], Kaela Kelly[1++], Jonathan M. Brotchie[2], James B. Koprich[2], Andrew B. West[1*]

[1]Duke Center for Neurodegeneration Research, Department of Pharmacology and Cancer Biology, Duke University, Durham, North Carolina, USA

[2]Atuka Inc., Toronto, Ontario, Canada

[++] Equal contribution.

[*]C.A. Information: andrew.west@duke.edu, 3 Genome Court, Durham, North Carolina, 27710 USA

Supplemental Figure

Figure 1a

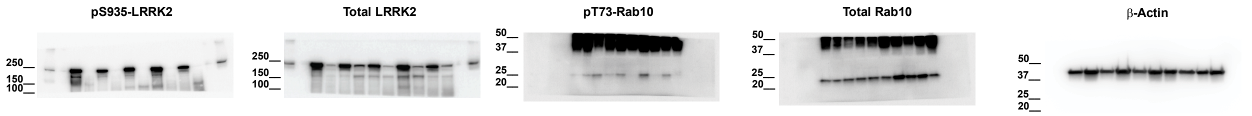

Figure 1b

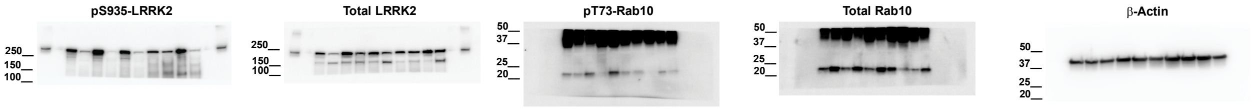

Figure 2a

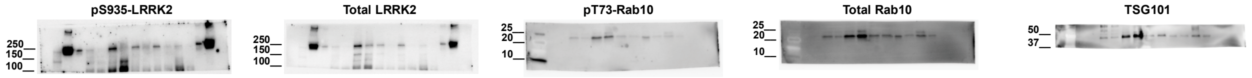

Figure 2b

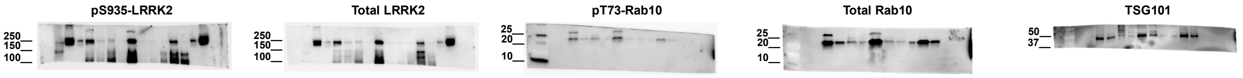

Figure 3a

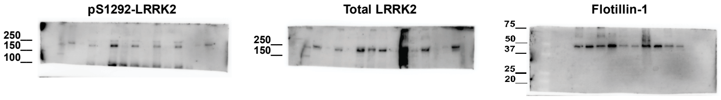

Figure 3b

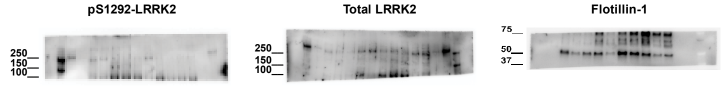

Supplemental Figure 1. Uncropped western blot images corresponding to the experiments in the indicated main figure panel.
