# Supplementary material for: Exosome markers of LRRK2 kinase inhibition
Source: NPJ Parkinsons Dis. 2020 Nov 13;6:32. doi: 10.1038/s41531-020-00138-7 (PMC7666125; doi:10.1038/s41531-020-00138-7)
Supplement: Supplementary file 2 — Reporting Summary [file 41531_2020_138_MOESM2_ESM.pdf]

nature research

Corresponding author(s): Andrew B. West

Last updated by author(s): 9/23/2020

Reporting Summary

Nature Research wishes to improve the reproducibility of the work that we publish. This form provides structure for consistency and transparency in reporting. For further information on Nature Research policies, see Authors & Referees and the Editorial Policy Checklist.

Statistics

For all statistical analyses, confirm that the following items are present in the figure legend, table legend, main text, or Methods section.

n/a	Confirmed	
☐	☒	The exact sample size (*n*) for each experimental group/condition, given as a discrete number and unit of measurement
☐	☒	A statement on whether measurements were taken from distinct samples or whether the same sample was measured repeatedly
☐	☒	The statistical test(s) used AND whether they are one- or two-sided<br>*Only common tests should be described solely by name; describe more complex techniques in the Methods section.*
☐	☒	A description of all covariates tested
☐	☒	A description of any assumptions or corrections, such as tests of normality and adjustment for multiple comparisons
☐	☒	A full description of the statistical parameters including central tendency (e.g. means) or other basic estimates (e.g. regression coefficient) AND variation (e.g. standard deviation) or associated estimates of uncertainty (e.g. confidence intervals)
☐	☒	For null hypothesis testing, the test statistic (e.g. *F*, *t*, *r*) with confidence intervals, effect sizes, degrees of freedom and *P* value noted<br>*Give P values as exact values whenever suitable.*
☒	☐	For Bayesian analysis, information on the choice of priors and Markov chain Monte Carlo settings
☒	☐	For hierarchical and complex designs, identification of the appropriate level for tests and full reporting of outcomes
☐	☒	Estimates of effect sizes (e.g. Cohen's *d*, Pearson's *r*), indicating how they were calculated

Our web collection on statistics for biologists contains articles on many of the points above.

Software and code

Policy information about availability of computer code

Data collection	N/A
Data analysis	N/A

For manuscripts utilizing custom algorithms or software that are central to the research but not yet described in published literature, software must be made available to editors/reviewers. We strongly encourage code deposition in a community repository (e.g. GitHub). See the Nature Research guidelines for submitting code & software for further information.

Data

Policy information about availability of data

All manuscripts must include a data availability statement. This statement should provide the following information, where applicable:

- Accession codes, unique identifiers, or web links for publicly available datasets
- A list of figures that have associated raw data
- A description of any restrictions on data availability

The datasets generated and/or analyzed during the current study are available from the corresponding author on reasonable request.

Field-specific reporting

Please select the one below that is the best fit for your research. If you are not sure, read the appropriate sections before making your selection.

☒ Life sciences ☐ Behavioural & social sciences ☐ Ecological, evolutionary & environmental sciences

For a reference copy of the document with all sections, see nature.com/documents/nr-reporting-summary-flat.pdf

Life sciences study design

All studies must disclose on these points even when the disclosure is negative.

Sample size	Sample sizes for the different biomarkers utilized were based on effect sizes and standard deviations obtained previously in rat and mouse models (see PMID 30048714, 25653221). Our group sizes greater or equal than 4 per treatment group in the study were 90% powered to detect a 50% difference in means for the biomarker, with a standard deviation of 20%.
Data exclusions	One value from one subject for phospho- and total Rab10 obtained from PBMC lysates from a treated group (PFE-360) was excluded for analysis because the total Rab10 detection fell below our limit of detection at a baseline collection. Additionally, urine analysis of RA283 was not available from one subject because the urine was not successfully collected from that subject. These exclusions are indicated in the results text and relevant legends.
Replication	Observations were made with two structurally independent molecules targeting the same ATP-binding pocket in the LRRK2 protein kinase. Technical repeats were completed at least twice for every measurement of every sample, with some samples measured more than twice depending the volume of specimens from the subjects available for analysis. Attempts at replication were considered successful.
Randomization	Samples were collected at baseline and after treatment. In biomarker measurements, samples were processed and proteins and lipids measured without subject group assignment.
Blinding	Investigators were blinded sample group identity until after final data curation.

Reporting for specific materials, systems and methods

We require information from authors about some types of materials, experimental systems and methods used in many studies. Here, indicate whether each material, system or method listed is relevant to your study. If you are not sure if a list item applies to your research, read the appropriate section before selecting a response.

Materials & experimental systems

n/a	Involved in the study	
☐	☒	Antibodies
☒	☐	Eukaryotic cell lines
☒	☐	Palaeontology
☐	☒	Animals and other organisms
☒	☐	Human research participants
☒	☐	Clinical data

Methods

n/a	Involved in the study	
☒	☐	ChIP-seq
☒	☐	Flow cytometry
☒	☐	MRI-based neuroimaging

Antibodies

Antibodies used	anti-LRRK2 (N241A/34, Antibodies Inc, Cat # 75-253), anti-LRRK2 (clone MJFF2 c41-2, Abcam, Cat # ab133474), anti-pS935 LRRK2 (clone UDD2-10, Abcam, Cat # ab133450), anti-pS1292-LRRK2 (clone MJFR-19-7-8, Abcam, Cat # ab203181), anti-pT73 Rab10 antibody (clone MJF-R21, Abcam, Cat # ab230261), anti-Rab10 (clone D36C4, Cell Signaling, Cat # 8127), anti-Flotillin-1 (clone D2V7J, Cell Signaling, Cat # 18634), and anti-TSG101 (ab30871, Abcam, Cat # ab30871). The following secondary antibodies were used: HRP conjugated donkey anti-rabbit secondary antibody (Jackson ImmunoResearch, # 711-035-152) and HRP conjugated donkey anti-mouse secondary antibody (Jackson ImmunoResearch, # 715-035-151).
Validation	Antibodies were validated for this study using recombinant protein controls on Western blot. They have also been used and validated with knockout/knockdown tissues and cells in previous studies (see PMIDs 26865512 , 27297049, 29166931).

Animals and other organisms

Policy information about studies involving animals; ARRIVE guidelines recommended for reporting animal research

Laboratory animals	Cynomolgus macaques
Wild animals	N/A
Field-collected samples	N/A
Ethics oversight	All husbandry, housing and experimental procedures were conducted at Suzhou Xishan Zhongke Drug R&D Co., Ltd. (Suzhou, PRC) and under an IACUC animal use protocol specific for this study and approved by Suzhou Xishan Zhongke Drug R&D Co., Ltd.. All methods were performed in accordance with the relevant guidelines and regulations.

Note that full information on the approval of the study protocol must also be provided in the manuscript.
